# The Distribution of Dengue Virus Serotype in Quang Nam Province (Vietnam) during the Outbreak in 2018

**DOI:** 10.3390/ijerph19031285

**Published:** 2022-01-24

**Authors:** Duong Q. Phan, Linh D. N. Nguyen, Son T. Pham, Tai Nguyen, Phuong T. T. Pham, Suong T. H. Nguyen, Dien T. Pham, Huong T. Pham, Duy K. Tran, Sa H. Le, Tung T. Pham, Kieu C. D. Nguyen, Gianna Dipalma, Alessio Danilo Inchingolo, Prisco Piscitelli, Alessandro Miani, Scacco Salvatore, Stefania Cantore, Sergey K. Aityan, Andrea Ballini, Francesco Inchingolo, Ciro Gargiulo Isacco, Van H. Pham

**Affiliations:** 1 The General Hospital of Quang Nam, Xã Tam Hiệp 51000, Vietnam; bsphanquangduong@gmail.com (D.Q.P.); tainguyen.bvtwqn@gmail.com (T.N.); 2 Faculty of Medicine, Phan Chau Trinh University, Danang City 550000, Vietnam; linh.nguyendo@pctu.edu.vn (L.D.N.N.); phuong.ptt@pctu.edu.vn (P.T.T.P.); suong.nth@pctu.edu.vn (S.T.H.N.); dien.pt@pctu.edu.vn (D.T.P.); tungyduoc1218@gmail.com (T.T.P.); van.pham@pctu.edu.vn (V.H.P.); 3 School of Medicine, The University of Sydney, Sydney, NSW 2006, Australia; truongson.pham@gmail.com; 4 Pham Ngoc Thach University of Medicine, Ho Chi Minh City 70000, Vietnam; phamthienhuong@gmail.com; 5 NK-Biotek Laboratories, Ho Chi Minh City 70000, Vietnam; khanhduytransh@gmail.com (D.K.T.); sa.shane.le@outlook.com (S.H.L.); 6 Department of Interdisciplinary Medicine, University of Bari “Aldo Moro”, 70124 Bari, Italy; drkieukaren@gmail.com (K.C.D.N.); giannadipalma@tiscali.it (G.D.); ad.inchingolo@libero.it (A.D.I.); stefaniacantore@pec.omceo.bari.it (S.C.); drciroisacco@gmail.com (C.G.I.); 7 Euro Mediterranean Scientific Biomedical Institute (ISBEM), 72023 Mesagne, Italy; 8 Italian Society of Environmental Medicine (SIMA), 20123 Milan, Italy; alessandro.miani@gmail.com; 9 Department of Environmental Science and Policy, University of Milan, 20132 Milan, Italy; 10 Department of Basic Medical Sciences, Neurosciences and Sense Organs, University of Bari “Aldo Moro”, 70124 Bari, Italy; salvatore.scacco@uniba.it; 11 Department of Multidisciplinary Research Centre, Lincoln University, Oakland, CA 94612, USA; aityan@lincolnuca.edu; 12 School of Medicine, University of Bari “Aldo Moro”, 70124 Bari, Italy; 13 Department of Precision Medicine, University of Campania “Luigi Vanvitelli”, 80138 Naples, Italy

**Keywords:** dengue hemorrhagic fever, dengue virus serotypes, Quang Nam, Vietnam

## Abstract

*Objectives*: Quang Nam province in the Centre of Vietnam has faced an outbreak of dengue hemorrhagic fever (DHF) in 2018. Although DHF is a recurrent disease in this area, no epidemiological and microbiological reports on dengue virus serotypes have been conducted mainly due to lack of facilities for such a kind of advanced surveillance. The aim of this study was to detect different dengue virus serotypes in patients’ blood samples. *Design and Methods*: Suspected cases living in Quang Nam province (Vietnam) and presenting clinical and hematological signs of dengue hemorrhagic fever were included in the study. The screening was performed, and the results were compared by using two methodologies: RT real-time PCR (RT-rPCR) and the Dengue NS1 rapid test. *Results*: From December 2018 to February 2019, looking both at RT-rPCR [+] and NS1 [+] methodologies, a total of 488 patients were screened and 336 were positive for dengue virus detection (74 children and 262 adults); 273 of these patients (81.3%) underwent viral serotype identification as follows: 12.82% (35/273) D1 serotype, 17.95% (49/273) D2, 0.37% (1/273) D3, 68.50 (187/283) D4, and 0.37% (1/273) D2+D4 serotypes. The RT-rPCR outcomes showed higher sensitivity during the first three days of infection compared to NS1 (92.3% vs. 89.7%). The NS1 increased sensitivity after the first 3 days whilst the RT-rPCR decreased. *Conclusions*: Advanced surveillance with dengue virus serotypes identification, if performed routinely, may help to predict and prevent further DHF epidemics based on the exposure of the different serotypes during different periods that lead to the intensification of disease severity as a consequence of antibody-dependent enhancement (ADE).

## 1. Introduction

Dengue virus (DENV) is the main cause of dengue hemorrhagic fever (DHF), and it is transmitted by the *Aedes aegypti* (*A.* *aegypti*) mosquito, a typical breed of tropical and subtropical areas. Clinically, DHF is classified as (i) silent DHF without warning signs, (ii) DHF with warning signs (mucosal bleeding, lethargy, persistent vomiting, fluid accumulation, increasing hematocrit, and decreasing platelets, etc.), and (iii) severe DHF, which is characterized by severe plasma leakage, severe hemorrhagic events, and organ failure. The disease can progress from mild to severe and death [1,2]. According to the World Health Organization (WHO), 50–100 million DHF occur annually, corresponding to a 30-fold increase compared to five decades ago [3,4]. In 2009, the number of infections dramatically increased with the DHF outbreaks in many countries in tropical and subtropical regions, including North and South America, Southern Europe, and especially in Southeast Asian Countries such as Vietnam [3,5]. In Vietnam, four DENV serotypes were detected linked to different outbreaks every year [6]. As reported by NhaTrang Pasteur Institute in 2018, about 17,200 DHF cases were diagnosed in 11 cities from Quang Binh to Binh Thuan and Quang Nam provinces. In the first 6 months of 2019, a total of 96,000 cases were reported in Vietnam, showing a three-fold increase compared to the previous year, with a mortality rate of 0.007% [7]. According to the report of Quang Nam Center for Disease Control, during the first 7 months of 2019, the number of DHF increased by three-fold in comparison to 2018 [8]. The DENV was classified into four serotypes: DENV-1, DENV-2, DENV-3, and DENV-4 [1,3]. The different serotypes could influence manifestation as well as the severity of DHF [9], resulting in increased risk if patients are re-infected with DENV-2 or DENV-3 compared to DENV-4 [10]. Data from Cuba, Hawaii, and Thailand revealed a similar figure: patients affected by DENV-1 showed a higher risk of being affected by severe DHF in case of secondary infections with DENV-2 or DENV-3 serotypes [11]. A different study on DHF conducted in Vietnam demonstrated that the clinical patterns of re-infected patients worsen when DENV-2 is followed by DENV-1 re-infection; then if DEN V-4 is followed by DENV-1; when DENV-3 is followed by DENV-2, and finally if DENV-3 is followed by DENV-4 [12].

The antibody-dependent enhancement (ADE) was applied to explain why under 1-year-old infants infected with a DENV serotype different from that of the mother showed a higher risk of developing severe DHF [13]. These data suggest that the detection of prevalent DENV serotypes is very important to help clinicians in treating the disease, as well as to assess more specific measures according to an accurate epidemiological assessment.

## 2. Materials and Methods

This was a cross-sectional study. Patients enrolled in the study were all subjects clinically diagnosed with DHF and hospitalized at Quang Nam General Hospital, in accordance with decision number 1499 issued on May 17th, 2011, by the Ministry of Health. Patients enrolled were living in or coming from an area with an outbreak or endemic of DHF within 14 days with sudden onset of high fever for 2–7 days and at least 2 of the following signs: (1) hemorrhagic signs at different levels: positive tourniquet test, cutaneous petechiae/purpura, gum or nose bleeding; (2) headache, loss of appetite, and nausea; (3) skin congestion, rash, or both; (4) muscular pain, joint pain, and orbital pain; (5) irritability and lethargy; (6) abdominal pain localized on the hypochondriac region. Each enrolled patient was given informed consent to sign in accordance with the revisited 2013 Helsinki Declaration. Blood samples were collected with 2–3 mL using EDTA-containing tubes.

Each sample was given a status including age, date of birth, gender, and the starting day of fever, together with the details regarding epidemic information. After collection, the blood samples were stored in the refrigerator (2–8 °C) for up to 8 h and sent to the Research Center of Phan Chau Trinh University in the foam box with gel ice packs. At the Research Center, the blood samples were immediately centrifuged at 3000 RPM for 10 min to isolate blood plasma and immediately analyzed by one-step reverse transcriptase real-time PCR (RT-rPCR) for DENV detection and for viral serotype identification that was referred from Gilberto A. Santiago (CDC) et al. [14,15]. Briefly, nucleic acids were extracted completely from 200µL plasma on the KingFisher FLEX system (Thermo, Waltham, MA, USA) using ^NK^DNARNAprep-MAGBEAD extraction kits manufactured by Nam Khoa company [16,17]. Extracted fluid (5 µL) was added into PCR 0.1 tubes of Rotor Gen Q MDx 5plex Platform (Qiagen, Kuala Lumpur, Malaysia), containing 20 µL one step RT rPCR mix made from Thermo AgPath-ID™ One-Step RT-PCR (Thermo, USA) with four specific primer pairs and four specific probes for four different DENV serotypes, that were: D1-F (CAA AAG GAA GTC GTG CAA TA), D1-R (CTG AGT GAA TTC TCT CTA CTG AAC), D1-PR (FAM-CAT GTG GTT GGG AGC ACG C-BHQ1) for DEN-1; D2-F (CAG GTT ATG GCA CTG TCA CGA T), D2-R (CCA TCT GCA GCA ACA CCA TCT C), D2-PR (HEX-CTC TCC GAG AAC AGG CCT CGA CTT CAA-BHQ1) for DENV-2; D3-F (GGA CTGG ACA CAC GCA CTC A), D3-R (CAT GTC TCT ACC TTC TCG ACT TGT CT), D3-PR (TexasRED-ACC TGG ATG TCG GCT GAA GGA GCT TG-BHQ2) for DENV-3 and; D4-F (TTG TCC TAA TGA TGC TGG TCG), D4-R (TCC ACC TGA GAC TCC TTC CA), D4-PR (CY5-TTC CTA CTC CTA CGC ATC GCA TTC CG-BHQ3) for DENV-4. All the primers and probes were ordered from Proligo (Sigma, Singapore). After this step, the PCR tubes were incubated in the Rotor Gen Q instruments (Qiagen, Malaysia) and ran with thermal cycles at 45 °C for 10 min to reverse transcriptase (RT), at 95 °C for 10 min to destroy the RT enzyme, 40 cycles with two thermal steps at 95 °C for 15 s followed by 60 °C for one minute in which we recorded fluorescent signals. The results were read and characterized together with DENV serotypes based on amplifying signals dyed with four different color channels: FAM for type 1, HEX for type 2, TexasRED for type 3, and CY5 for type 4. If signals were absent in all channels, we ran another assay using RT-rPCR mix manufactured by Nam Khoa company for house-keeping gene, RNAseP, to confirm the negativity of the sample

The enrolled patients were also provided a dengue NS1 rapid test by the laboratory of the Quang Nam Region General Hospital. This test was based on the immunochromatography lateral flow that could detect the NS1 antigen of DENV circulated in the blood of the patients during the infection, as per manufacturer description (SD BIOLINE DENGUE NS1 AG, SD manufacturer).

For statistical analyses, the SPSS 17.0 statistical software was used as a statistical procedure for the analysis of these data. The Chi-square procedure was used for all counting materials and a t-test for measurement material. Laboratory data were then compared between the groups using single-factor ANOVA analysis (*p* < 0.05 was considered statistically significant).

## 3. Results

**Figure 1:**
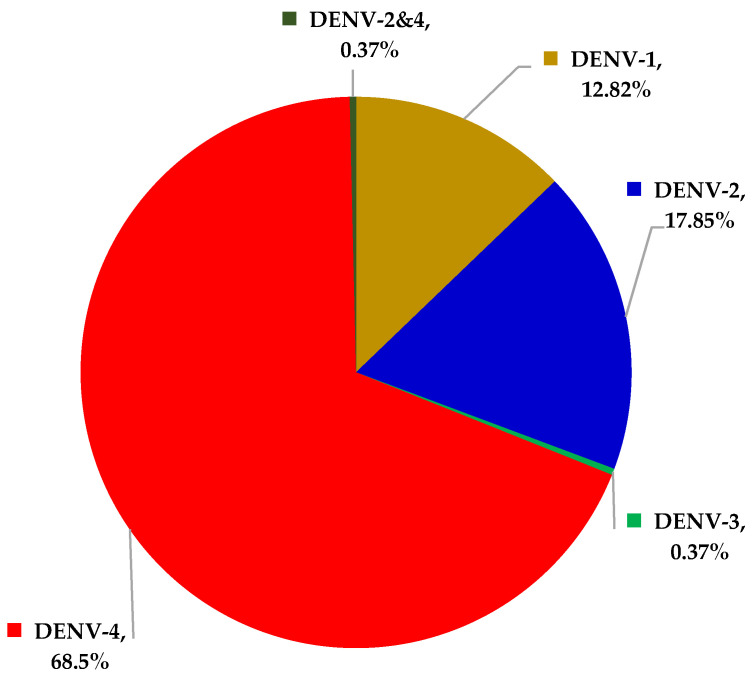
Percentages of DENV serotypes among 273 patients who tested positive for DENV by RT-rPCR.

From December 2018 to February 2019, 488 patients were enrolled in the study, including 111 children and 377 adults. Among these patients, 336, including 74 children (22.02%) and 262 adults (77.98%), were diagnosed with DHF based on the positive results either from RT-rPCR or NS1 tests. The proportion of males and females in 74 pediatric patients were 59.46% (44/74) and 40.54% (30/74), respectively; among the 262 adults, 48.47% (127/262) were men and 51.53% (135/262) were women. The RT-rPCR detected DENV in 273 patients with the following different percentages of DENV serotypes: 12.82% (35/273) DENV-1, 17.95% (49/273) DENV-2, 0.37% (1/273) DENV-3, and 68.50% (187/273) DENV-4. There was one case (0.37%) of co-infection of DEN-2 with DENV-4. The percentages of DENV serotypes in our study are shown on the Figure 1.

The analysis of the RT-rPCR and the NS1 results showed that among 336 confirmed patients affected by DHF, 273 tested positive by RT-rPCR, and 304 tested positive with NS1. The results of both the RT-rPCR and the NS1 tests on 488 participants are resumed in Table 1.

The sensitivity in the first 3 days of the diseases of the RT-rPCR assay and NS1 test in the diagnosis of DHF described in this study (Table 2) showed around 92.3% (108/117) and 89.7% (105/117) values, respectively. However, these differences were not statistically significant (t = 0.686, *p* > 0.05). From day 4 to 9 of the disease, the sensitivity of RT-rPCR gradually decreased from 82% to 33% on day 8; the test was not able to detect DENV on day 9. The average sensitivity of the test during this time was 75% (141/188). On the contrary, the average sensitivity of the NS1 test was continuously high (89.9% = 169/188) from day 4 and even on day 9.

The difference between the sensitivity of the RT-rPCR assay and the NS1 rapid test was statistically significant (t = 3.796, *p* < 0.05). Table 2 shows the sensitivity of the RT-rPCR and the NS1 in the diagnosis of DHF per day of infection. The sensitivity of the test per day of infection was defined by the percentage of the DHF cases detected by the test on the overall DHF cases. For example, among 85 cases of DHF with D4 infection, 70 were detected by RT-rPCR and 76 were detected by NS1; then, the sensitivity of RT-rPCR was 82.35% (70/85), and the sensitivity of NS1 was 89.41% (76/85)

As shown in Table 3, most clinical manifestations in the 336 DHF patients were fever, headache, orbital pain, and joint pain, which occurred in 80–90% of the patients. Among these manifestations, orbital pain and joint pain appeared more frequently in adults than children. The explanation for this difference may be due to children’s behavior that usually tends not to show this type of malaise. Lethargy and increased liver enzymes were the second-highest symptom manifestation in about 40–45% of the patients. Hematological findings such as Hematocrit (Hct) > 42%, thrombocytopenia, and cutaneous hemorrhage were present in about 22–33% of the patients. Gastrointestinal (GI) bleeding, hematuria, fluid accumulation detected by ultrasounds, and thrombocytopenia < 20,000 were rare events. More than half of the patients (56.25%) were classified as DHF with warning signs, manifesting at least one of these signs: lethargy, mucous membrane hemorrhages, liver enzyme increase, Hct increase, and rapid platelet decrease. However, there was no one classified as severe DHF, probably because severe DHF patients were referred to higher-level hospitals (often located in the big cities outside the rural area under investigation) before the study was conducted. Apart from subjective manifestations, there was no other difference in clinical settings between children and adults diagnosed with DHF in our study.

Table 4 shows clinical manifestations and subclinical findings associated with different DENV serotypes in patients diagnosed with DHF. The table only shows DENV-1, -2, and -4 because DENV-3 was detected in a few cases, and DENV-2 and -4 co-infection was detected merely in one patient. As the results show in Table 4, in our study, there were almost similar clinical manifestations and subclinical findings various among DENV serotypes. These results could be explained by the absence of severe DHF cases; thereby, we were not able to analyze the difference in clinical manifestations and subclinical findings among DENV serotypes in our DHF patients. The outcomes also revealed a small number of gastrointestinal bleedings (2.22%), severe thrombocytopenia < 10,000, and abdominal fluid retention (8.33%), features that were observed only in patients infected with DENV-2. These manifestations were very rare events in patients diagnosed with different DENV serotypes.

## 4. Discussion

While Asian countries often experience DHF outbreaks, which rarely affect children under 15 of age, in South America, DHF widely spreads among the population independently from the age, probably due to different lifestyles [18,19,20,21]. In this study, 77.98% of infected patients were adults, more than three folds compared to children (22.02%), with data statistically significant (t = 14.505, *p* < 0.001).

An individual primarily infected with DENV could create specific antibodies for the primary serotype; however, these antibodies not only could not be enough to fight against other serotypes but also trigger clinical manifestations when patients are secondarily infected with other DENV serotypes. It was probably because these useless antibodies increased the adhesive and invasive ability of DENV into growing cells, promoting the immune reaction through releasing inflammatory cytokines such as IL6, IL1, TNF, and IFN. The main consequence of this uncontrolled inflammatory condition is the increased permeability of vessels and blood coagulation, as proposed by the theory of antibody-dependent enhancement [22] in the physiopathology of DHF. Although the theory has not demonstrated this so far, it was the only explanation for the physiopathology of DHF and the ability to cause the outbreak of the DHF when the distribution of DENV serotypes change in various regions. Cummings et al. reported DENV serotypes changes in cycles of 8–10 years in Thailand [23]. From 1980 to 2000, Adam et al. also showed that DHF epidemics were caused by a rotation of three DENV serotypes: DENV-1, DENV-2, and DENV-3 [24]. The assumption could be based on two reasons. First, infected individuals may generate antibodies explicitly against only one serotype while remaining vulnerable to others, making new outbreaks always an open possibility. Second, the different serotypes are always available in mosquitos; thus, they not only endemically maintain the disease but also ready make the outbreak with new serotypes.

The outcomes of DENV serotype analysis in Vietnam, obtained during the largest DHF outbreak in 1987, showed 83,905 infections and 904 deaths mainly caused by DENV-2 (90.5%). On the other hand, in 1990, DENV-1 appeared and soon spread widely, reaching 62.5% contagious during 1993. DENV-3 was also detected with a lower proportion during the same period. From 1994 to 1998, scientists isolated the DENV-3, which showed the following decrease of DENV-1 distribution. In 1998, the prominent DENV serotype isolated was again the DENV-3 [25]. Across the years, the DENV-3 steadily decreased until it was completely replaced by other serotypes in 2000. Our findings showed that DENV-4 (68.5%) was prominent in the recent DHF outbreak in Quang Nam province (2018), followed by DENV-2 (17.9%), DENV-1 (12.8%), and DENV-3 (1 case, 0.4%).

When comparing data from studies conducted in HCM city in four consecutive years, from 2010 to 2013 [26], we could find the prominent distribution of DENV-1 (36%), followed by DENV-4 (27%), DENV-2 (26%), and DENV-3 (11%). While a different study conducted in Dong Thap province during the same period [27] from February 2012 to February 2013, the outcomes showed a different scenario: the DENV-2 distribution was the highest (36%), followed by DENV-4 (35%), DENV-3 (19%), and DENV-1 (11%). Based on the data provided above, we could recognize that the prominent distribution of DENV serotypes was likely to differ between regions but similar in trend of the increasingly prominent distribution of DENV-4 and the gradually decreasing of DENV-3. Therefore, longitudinal studies for the distribution of DENV serotypes are necessary for predicting the DHF outbreaks in a certain region and country. These studies will also be the trend of our DHF research in the future.

In addition, several studies revealed the relationship between DENV serotypes and the severity of DHF [10,11,12,13], showing a strict bond existing between the virus, host, and the environment. By this mechanism, the severity of DHF outbreak could be predicted based on the identification of DENV serotype characterized in the previous outbreak [11,12]. This aspect could be the main topic of investigation in future studies as a starting point for the creation of a specific algorithm and for the development of a more precise diagnostic approach based on targeting antibodies for each of the DENV serotypes.

At the time of this article, the antibody (IgG or IgM) assays represented valuable tools in diagnosis whether patients were facing a primary or secondary infection. NS1 tests were highly valuable as rapid tests based on immune chromatography, which is an easily assessed procedure in laboratories or even on-site in the clinical wards [28]. There are many manufacturers that can provide the basic kits needed to perform these tests that can reach a specificity as higher as 90% with a grade of high sensitivity that can be stable for the first three days [28]. The NS1 tests performed in Region General Hospital of Quang Nam province were highly sensitive, like the RT-rPCR assay in the first three days, and were stable until day 9 of the condition. However, due to the limitation of blood samples on day 8 and day 9, it was difficult to consolidate these results.

## 5. Conclusions

Although DHF outbreaks are worsening in Quang Nam province with the increasing numbers of infections, studies describing the distribution of DENV serotypes are rare. Assessing the circulation and the grade of transmission of DENV serotypes could help local health authorities in predicting new foci of infection, thus allowing them to implement proper treatment strategies at a local level and nationwide. Owing to the application of RT-rPCR assay in detecting and serotyping DENV in patients’ blood samples, our study could be helpful in elucidating the question regarding which of the DENV serotype would be prominent during the next DHF outbreak. We plan to continue the study in the next years to better describe the DHF epidemiology in Vietnam.

## Figures and Tables

**Table 1:** Results of qPCR assay and NS1 tests in the overall 488 participants.

		One-Step RT-rPCR		Total
		[+]	[−]	
NS1	[+]	241	63	304
	[−]	32	152	184
Total		273	215	488

**Table 2:** The sensitivity of RT-rPCR and NS1 in diagnosis of DHF per day of infection.

Day of Infection	DHF Case	NS1(+) Case	RT-rPCR(+) Case	Sensitivity of NS1	Sensitivity of RT-rPCR
D9	1	1	0	100.00% (1/1)	0.00% (0/1)
D8	3	3	1	100.00% (3/3)	33.33% (1/3)
D7	11	10	7	90.91% (10/11)	63.64% (7/11)
D6	28	24	16	85.71% (24/28)	57.14% (16/28)
D5	60	55	47	91.67% (55/60)	78.33% (47/60)
D4	85	76	70	89.41% (76/85)	82.35% (70/85)
D3	75	68	71	90.67% (68/75)	94.67% (71/75)
D2	38	34	33	89.47% (34/38)	86.84% (33/38)
D1	4	3	4	75.00% (3/4)	100.00% (4/4)
D0	0	0	0		
Undetermined	31	30	24	96.77% (30/31)	77.42% (24/31)
Total	336	304	273	90.48% (304/336)	81.25% (273/336)

**Table 3:** Proportion of clinical manifestations in 336 patients diagnosed with DHF.

	Children	Adults	Total
Fever	93.15	92.09	92.33
Orbital pain	79.71	90.28	87.97
Muscular/joint pain	68.12	86.59	82.54
Positive tourniquet test	0	0.82	0.63
Cutaneous hemorrhages	25.00	21.63	22.36
Gum bleeding	13.24	15.10	14.70
Mucous membrane hemorrhages	2.94	2.04	2.24
Lethargy	35.29	47.76	45.05
Abdominal pain	2.70	0.78	1.21
Hepatomegaly	0	0	0
Hct increase > 42%	24.66	28.19	27.41
Platelet decrease < 20 K	2.70	7.72	6.61
Platelet decrease < 100 K	25.68	35.91	33.63
DHFD with warning signs	51.35	57.63	56.25
Liver enzyme increase	33.78	41.31	39.64
Abdominal fluid accumulation	0	2.32	1.80

**Table 4:** Clinical manifestations and subclinical findings associated with different DENV serotypes in patients diagnosed with DHF.

	DENV-1	DENV-2	DENV-4
Sustained fever	63.64	72.34	69.73
Fever	83.33	92.31	78.57
Orbital pain	78.13	88.89	91.67
Muscular/joint pain	78.13	88.89	81.01
Positive tourniquet test	0	0	1.12
Cutaneous hemorrhages	31.25	11.11	17.42
Gum bleeding	15.63	11.11	14.04
Subconjunctival hemorrhage	0	0	0.56
Hematuria	3.13	0	0
GI bleeding	0	2.22	0
Lethargy	46.88	37.78	47.75
Abdominal pain	0	2.08	1.62
Hepatomegaly	0	0	0
Hct ≥ 42%	2.94	12.5	9.14
PLT < 10,000	0	4.17	0
PLT 10,000–20,000	0.03	0.06	0.06
PLT 20,000–100,000	44.12	39.58	29.41
DHFD with warning signs	48.57	59.18	59.36
Liver enzymes > 40–< 400	50	37.5	36.02
Abdominal fluid (ultrasounds)	0	8.33	1.61

## Data Availability

Data can be accessed upon request to van.pham@pctu.edu.vn (V.H.P.).
